# When more isn’t better: the surprising superiority of glycoprotein-only Marburg vaccines

**DOI:** 10.1172/JCI195933

**Published:** 2025-09-02

**Authors:** Pablo Penaloza-MacMaster

Marburg virus (MARV) is classified by the WHO as a priority pathogen due to its high fatality rate and potential to cause outbreaks. Despite the evaluation of various MARV vaccine candidates, there is currently no licensed vaccine, and the optimal antigen formulation for achieving protective immunity remains unclear.

Virus-like particle–based (VLP-based) vaccines represent a versatile and effective platform that has been successfully implemented in several clinically approved vaccines, including those against human papillomavirus (HPV), hepatitis B virus (HBV), and hepatitis E virus (HEV). VLPs can self-assemble into nanostructures that mimic the morphology of authentic viruses but lack replicative genetic material, rendering them noninfectious. By recapitulating the structural features of native virions, VLPs can elicit antigenically relevant immune responses, including neutralizing antibodies that recognize conformational epitopes.

In addition to the surface glycoprotein (GP), VLPs can be engineered to include other structural viral antigens, with the aim of broadening immune responses and potentially enhancing protection beyond what is achieved with vaccines targeting only a single antigen. For example, studies conducted by my research group and others showed that SARS-CoV-2 vaccines expressing both spike and nucleoprotein are more protective than vaccines expressing either antigen alone (1, 2), suggesting that multiantigen formulations can potentiate vaccine efficacy. VLPs comprising multiple antigens are being explored for various pathogens like HIV (3) and filoviruses (4, 5), but whether inclusion of multiple antigens universally enhances vaccine efficacy remains an open question.

In this issue of the *JCI*, a study by Subramani and colleagues compared the protective efficacy of a MARV mRNA vaccine encoding only the GP with a vaccine encoding both the GP and the VP40 matrix protein, which self-assemble to form VLPs (6). At high vaccine doses, both vaccines conferred 100% protection against lethal MARV challenges. However, at low vaccine doses, the GP-only vaccine maintained full protection, whereas the VLP-based vaccine expressing both GP and VP40 conferred only partial protection. These findings demonstrate that under suboptimal dosing conditions, a multicomponent vaccine encoding both GP and VP40 is less effective than a monovalent GP-only vaccine.

This study also underscores the importance of empirically validating assumptions about antigenic breadth in multicomponent vaccine formulations. While VLPs offer structural and immunological advantages that can broaden immune responses to multiple epitopes, the inclusion of additional viral antigens beyond the surface GP does not universally improve protective immunity and, in some cases, may even interfere with other responses. These results warrant a reevaluation of VLP vaccine design for Marburg and other filoviruses and potentially for additional viruses for which similar assumptions about the superiority of VLPs have been made.

## Version 1 09/02/2025

Electronic publication
